# Injury Causes and Severity in Pediatric Traumatic Brain Injury Patients Admitted to the Ward or Intensive Care Unit: A Collaborative European Neurotrauma Effectiveness Research in Traumatic Brain Injury (CENTER-TBI) Study

**DOI:** 10.3389/fneur.2020.00345

**Published:** 2020-04-30

**Authors:** Lennart Riemann, Klaus Zweckberger, Andreas Unterberg, Ahmed El Damaty, Alexander Younsi, Collaborative European NeuroTrauma Effectiveness Research in Traumatic Brain Injury (CENTER-TBI) Investigators and Participants

**Affiliations:** Department of Neurosurgery, University Hospital Heidelberg, Heidelberg, Germany

**Keywords:** pediatric TBI, children, traumatic brain injury, injury characteristics, CT imaging, outcome

## Abstract

**Background:** Traumatic brain injury (TBI) is the leading cause of death and disability in children. It includes a range of different pathologies that differ considerably from adult TBI. Analyzing and understanding injury patterns of pediatric TBI is essential to establishing new preventive efforts as well as to improve clinical management.

**Methods:** The multi-center, prospectively collected CENTER-TBI core and registry databases were screened and patients were included when younger than 18 years at enrollment and admitted to the regular ward (admission stratum) or intensive care unit (ICU stratum) following TBI. Patient demographics, injury causes, clinical findings, brain CT imaging details, and outcome (GOSE at 6 months follow-up) were retrieved and analyzed. Injury characteristics were compared between patients admitted to the regular ward and ICU and multivariate analysis of factors predicting an unfavorable outcome (GOSE 1-4) was performed. Results from the core study were compared to the registry dataset which includes larger patient numbers but no follow-up data.

**Results:** Two hundred and twenty seven patients in the core dataset and 687 patients in the registry dataset were included in this study. In the core dataset, road-traffic incidents were the most common cause of injury overall and in the ICU stratum, while incidental falls were most common in the admission stratum. Brain injury was considered serious to severe in the majority of patients and concurrent injuries in other body parts were very common. Intracranial abnormalities were detected in 60% of initial brain CTs. Intra- and extracranial surgical interventions were performed in one-fifth of patients. The overall mortality rate was 3% and the rate of unfavorable outcome 10%, with those numbers being considerably higher among ICU patients. GCS and the occurrence of secondary insults could be identified as independent predictors for an unfavorable outcome. Injury characteristics from the core study could be confirmed in the registry dataset.

**Conclusion:** Our study displays the most common injury causes and characteristics of pediatric TBI patients that are treated in the regular ward or ICU in Europe. Road-traffic incidents were especially common in ICU patients, indicating that preventive efforts could be effective in decreasing the incidence of severe TBI in children.

## Introduction

Traumatic brain injury (TBI) is considered to be the leading cause of death and disability in children (1, 2). Neurological and psychological deficits resulting from TBI can be a high burden for patients and their relatives, deeply affecting the child's physical, cognitive and behavioral development. Pediatric TBI is a global problem with high incidence numbers reported from both, developed and developing countries including the United States, Europe, Iran, and India (3). In the United States alone, it accounts for more than 1 billion US-Dollars in hospital charges every year (1). While pediatric TBI encompasses a wide range of traumatic brain pathologies, it differs from adult TBI in terms of pathophysiology, injury causes, and management. It is therefore important to recognize pediatric TBI as an own entity and study injury characteristics separately from adult TBI. Analyzing and understanding injury patterns of pediatric TBI is essential to finding and establishing new preventive efforts and public campaigns as well as to improve clinical management. Prevention plays a critical role in pediatric TBI, as the vast majority of brain injuries in children occur unintentionally. The multi-center CENTER-TBI study provides the opportunity to analyze injury patterns, clinical characteristics, and radiological findings in pediatric patients from many countries across Europe. We hope that insights from our study can help to better understand pediatric TBI, reflect on current clinical care, and provide information to shape targeted preventive efforts to reduce the incidence of this very serious condition.

## Materials and Methods

### Study Design and Patient Selection

**Figure 1:**
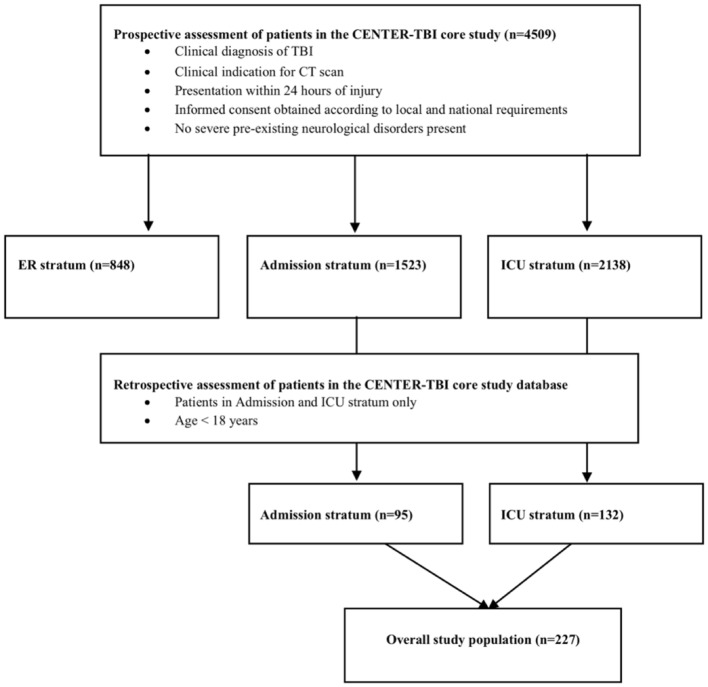
Flow-Diagram of patient selection within the CENTER-TBI core study (separate from the CENTER-TBI registry).

For the present analysis, data from the Collaborative European NeuroTrauma Effectiveness Research in Traumatic Brain Injury (CENTER-TBI) core study and the CENTER-TBI registry were used. The CENTER-TBI core study is a multi-center prospective longitudinal and observational cohort study conducted in Europe and Israel. Eligibility criteria for this study were a clinical diagnosis of TBI, presentation within 24 h of injury, an indication for brain CT scanning, and informed consent (see below) (4). Participants were recruited from December 2014 through December 2017 from 59 centers and enrolled in three strata, differentiated by care path: Emergency Room (ER) stratum (patients discharged from the ER), admission (ADM) stratum (patients admitted to the hospital ward), or Intensive Care Unit (ICU) stratum (patients admitted primarily to the ICU). Besides the core study, CENTER-TBI however also includes a registry with observational data of an even larger cohort of patients with TBI and an indication for brain CT scanning which is meant to permit validation and generalization of results from the core dataset but includes fewer variables and in particular no follow-up data such as Glasgow Outcome Scale extended (GOSE) ratings. The CENTER-TBI study protocol was approved by the national and local ethics committees for each recruiting site and informed consent by a legal representative/next of kin was obtained, according to local legislations, for all recruited pediatric patients. The sites, ethical committees, approval numbers, and approval dates are listed on the website: https://www.center-tbi.eu/project/ethical-approval. For the present study, all patients within the CENTER-TBI core and registry dataset were screened and included if they met the following inclusion criteria: (a) admission to either the regular ward (admission stratum) or the ICU (ICU stratum) and (b) age at presentation < 18 years (a flow-diagram of patient selection is provided in Figure 1).

### Data Collection

Our study was primarily conducted with the CENTER-TBI core dataset because it provides more variables and especially outcome-related data such as GOSE ratings in comparison to the CENTER-TBI registry. Where possible, we compared key results from the core dataset with the registry dataset to confirm findings in an even larger patient cohort. The following variables regarding patient demographics and injury causes were collected from the core dataset: age, sex, injury type, place, area, cause, and intention. To assess injury severity and clinical status at admission, the AVPU (Alert, Verbal, Pain, Unresponsive) status, Glasgow Coma Scale (GCS), GCS—motor score, pupillary response, total Injury Severity Score (ISS), and Abbreviated Injury Scale (AIS) for brain injury, head/neck, cervical/thoracic/lumbar spine, thorax, abdomen/pelvis, upper and lower extremities, and skin were retrieved. Radiological injury characteristics (e.g., presence of midline shift, epidural hematoma, acute or subacute subdural hematoma, subarachnoidal hemorrhage, intraventricular hemorrhage, contusion, traumatic axonal injury, cisternal compression, subdural collections/mixed density hematoma, mass lesion, skull fracture) as well as the Marshall and Rotterdam CT scores were obtained from initial brain CT scans. In terms of clinical care, the performance of surgical interventions (intra- and extracranial) was recorded. Secondary injury insults during the pre-hospital and emergency room phase were evaluated and included hypoxia (PaO_2_ <60 mmHg and/or SaO_2_ <90% or suspected by clinical signs such as cyanosis), hypotension (systolic blood pressure <90 mmHg, patients reported to be in shock and/or absent brachial pulse not related to extremity injury), hypothermia (documented core temperature <35°C), seizures (partial, generalized, status epilepticus), or cardiac arrest. The imputed six month Glasgow Outcome Scale Extended (GOSE) variable provided in the Neurobot database which includes both, observed ratings and imputed values was used to assess outcome. Values were only imputed if at least one GOSE rating from another time point was available per patient. Unfavorable outcome was defined as a GOSE score from 1 to 4 and a favorable outcome as a GOSE score from 5 to 8. From the registry dataset, the variables age, sex, injury type, place, cause, total ISS, AIS Brain Injury, GCS motor/verbal/eyes, and presence of abnormality on brain CT were obtained. All variables were retrieved from the CENTER-TBI Neurobot database (CENTER core version 2.0 and registry version 2.0).

### Statistical Analysis

Patient demographics, injury, and imaging characteristics as well as clinical data were summarized using descriptive statistics. Results are given as median + interquartile range (IQR) unless stated otherwise. For group comparisons, the Mann-Whitney *U* test was used for continuous variables while the chi-squared test was used for categorical variables. For outcome analysis, only patients with available GOSE ratings at six months were included. Multivariate logistic regression to an unfavorable outcome (GOSE 1-4) was performed with a model that included the predictors age, gender, road-traffic-incident (yes/no), GCS at admission, total ISS and secondary insults (yes/no). Hereby, missing data were addressed using complete case analysis. A *p*-value < 0.05 was considered statistically significant. No p value adjustment for multiple testing was performed due to the exploratory design of this study. All analyses were conducted with the statistical software R (5).

## Results

### Patient Cohort

In total, 227 TBI patients younger than 18 years from the CENTER-TBI core dataset were included in this study. Pediatric patients had been enrolled in 33 of the 59 participating centers. 95 (42%) of them were admitted to the hospital ward (admission stratum) while 132 (58%) patients required critical care and were admitted to the ICU (ICU stratum). The median age of the entire cohort was 14 (IQR 8–16, range 0–17) years and 64% of patients (*n* = 146) were males. Patient demographics were similar in the admission and ICU subgroup (Table 1).

**Table 1:** Injury causes- and details of pediatric TBI patients in the admission and ICU stratum of the CENTER-TBI core study.

**Characteristic**	**Total**	**Admission stratum**	**ICU stratum**	***p*-value**
Number of patients	227	95 (42%)	132 (58%)	-
Age (IQR)	14 (8-16)	13 (9-16)	14 (8-16)	0.583
Sex				0.695
- Female	81 (36%)	32 (34%)	49 (37%)	
- Male	146 (64%)	63 (66%)	83 (63%)	
Injury area				0.072
- Urban	153 (67%)	72 (76%)	81 (61%)	
- Rural	65 (29%)	20 (21%)	45 (34%)	
- Unknown	9 (4%)	3 (3%)	6 (5%)	
Injury intention				0.805
- Intentional	6 (3%)	3 (3%)	3 (2%)	
- Unintentional	212 (93%)	94%)	123 (93%)	
- Undetermined	9 (4%)	3 (3%)	6 (5%)	
Injury cause				**0.010**
- Road traffic incident	110 (48%)	33 (35%)	77 (58%)	
- Incidental fall	78 (34%)	41 (43%)	37 (28%)	
- Other non-intentional injury	22 (10%)	11 (12%)	11 (8%)	
- Violence	5 (2%)	4 (4%)	1 (1%)	
- Other	11 (5%)	5 (5%)	0 (0%)	
- Unknown	1 (0%)	1 (1%)	6 (5%)	
Injury road incidents				0.069
- Motor vehicle occupant	21 (19%)	3 (9%)	18 (23%)	
- Pedestrian	37 (34%)	11 (33%)	26 (34%)	
- Cyclist	27 (25%)	11 (33%)	16 (21%)	
- Scooter	13 (12%)	7 (21%)	6 (8%)	
- Motor Bike	10 (9%)	1 (3%)	9 (12%)	
- Other	2 (2%)	1 (3%)	2 (3%)	
Injury place				**0.015**
- Street/Highway	118 (52%)	38 (40%)	80 (61%)	
- Home	40 (18%)	19 (20%)	21 (16%)	
- School	14 (6%)	7 (7%)	7 (5%)	
- Sport/Recreation	40 (18%)	21 (22%)	19 (14%)	
- Public location	11 (5%)	8 (8%)	3 (2%)	
- Other	2 (1%)	0 (0%)	2 (2%)	
- Unknown	2 (1%)	2 (2%)	0 (0%)	
Total ISS (IQR)	18 (10-32)	10 (9-17)	26 (17-41)	**<0.001**
AIS Brain Injury	4 (3-4)	3 (3-3)	4 (4-5)	**<0.001**
Face injury	68 (30%)	28 (29%)	40 (30%)	1
Head/ Neck injury	104 (46%)	44 (46%)	59 (45%)	1
Cervical spine injury	12 (5%)	5 (5%)	7 (5%)	1
Thoracic spine injury	7 (3%)	3 (3%)	4 (3%)	1
Lumbar spine injury	4 (2%)	1 (1%)	3 (2%)	0.859
Thorax injury	55 (24%)	5 (5%)	50 (38%)	**<0.001**
Abdominal injury	16 (7%)	2 (2%)	14 (11%)	**0.027**
Pelvic injury	15 (7%)	3 (3%)	12 (9%)	0.133
Upper extremity injury	40 (18%)	18 (19%)	22 (17%)	0.788
Lower extremity injury	37 (16%)	10 (11%)	27 (20%)	0.069
Skin injury	31 (14%)	8 (8%)	23 (17%)	0.080

***AIS**, Abbreviated Injury Scale; **ISS**, Injury Severity Score; **IQR**, Interquartile range. Significant differences (p-value of <0.05 in the Mann-Whitney U test for continuous variables and chi-squared test for categorical variables) between the admission and ICU stratum are printed bold*.

### Injury Causes

**Figure 2:**
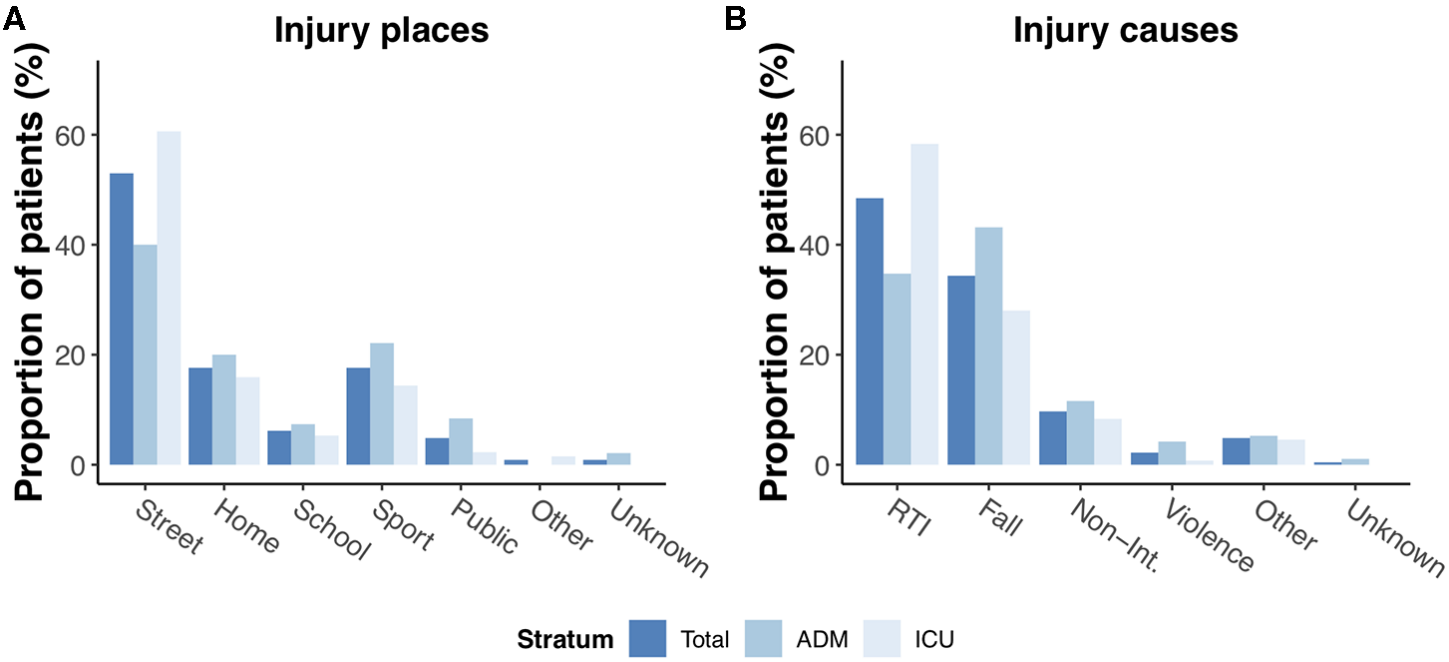
Injury places **(A)** and causes **(B)** of pediatric TBI in the entire core dataset and admission/ICU patients. RTI: Road-traffic incident; Non-Int.: Other non-intentional injury.

The most common places of injury overall were streets and highways (52%), especially in the ICU stratum where more than 60% of injuries occurred in that setting (Figure 2A). Injuries that occurred at home and sport/recreational places came second and accounted together for more than 40% of injuries in the admission stratum. Correspondingly, road traffic incidents were overall the most common cause of pediatric TBIs because of its high prevalence in the ICU stratum (58%), whereas incidental falls were the most common cause of injury in the admission stratum (43%, Figure 2B). In road-traffic incidents, the young patients were involved as pedestrians in one-third of cases (Table 1). Notably, more than half (52%) of all patients involved in an accident as cyclists, scooter drivers, or motor bikers did not wear a safety helmet.

### Injury Severity and Clinical Status at Admission

**Figure 3:**
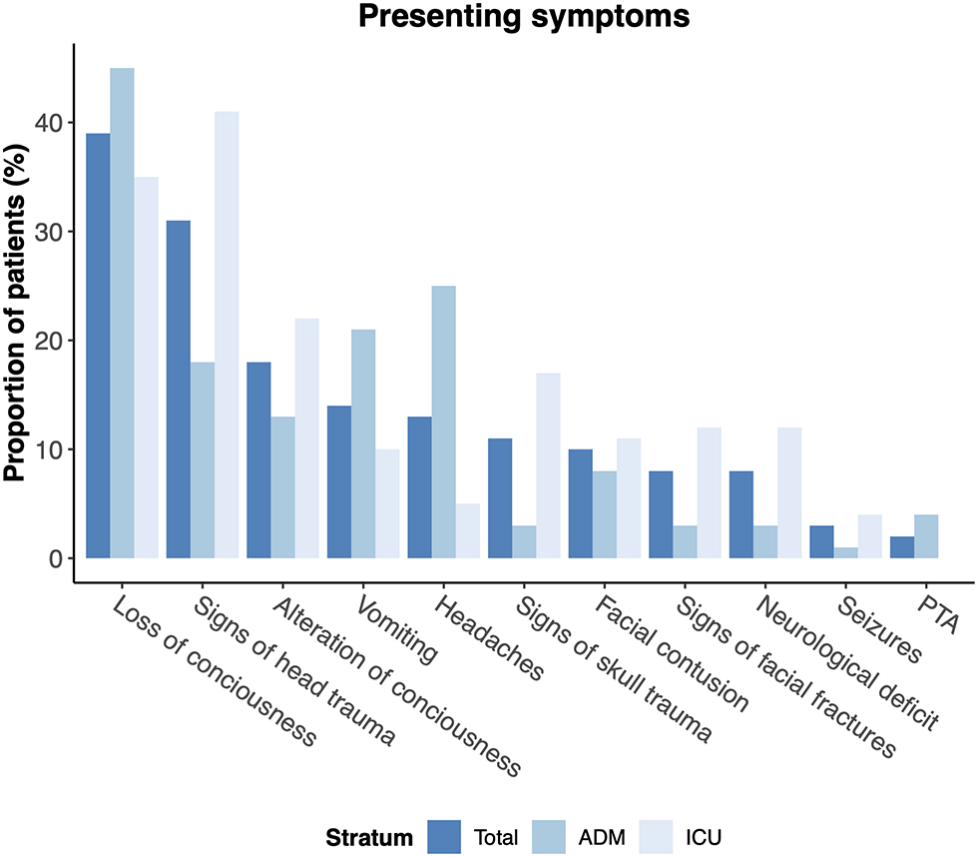
Presenting symptoms of pediatric patients with TBI. PTA: Post-traumatic amnesia.

Twenty two percent of pediatric patients in our cohort presented with mild TBI (GCS 13-15) while moderate (GCS 9-12) and severe TBI (GCS 3-8) occurred in 12 and 61%, respectively. Brain injury was considered serious to severe in the majority of patients (AIS 3 (3–4), Table 1). Concurrent injuries in other body regions were very common, especially in the ICU stratum. Overall, 176 patients (77%) presented with at least one concurrent injury. These mostly involved the face (30%) and head (46%), but also e.g., upper (18%) and lower extremities (16%). A simultaneous thorax injury was documented in 38% of all ICU patients as opposed to 5% in the admission stratum (*p* < 0.001). As expected, the total ISS and AIS Brain Injury scores in the ICU stratum were significantly higher than in the admission stratum (26 (17–41) vs. 10 (9–17), *p* < 0.001 and 4 (4–5) vs. 3 (3-3), *p* < 0.001, respectively). GCS as well as GCS—motor at admission were significantly higher in the admission stratum compared to the ICU stratum (15 (15–15) vs. 11 (6–14); *p* < 0.00; and 6 (6–6) vs. 5 (1–6); *p* < 0.001). In the ICU stratum, 28% of patients had no motor response to stimuli at all and one in ten patients had two unresponsive pupils at admission. The most common presenting symptoms are provided in Figure 3.

### Brain CT Imaging

In pediatric TBI patients within the core dataset, an intracranial abnormality was detected in more than 60% of all patients in the initial brain CT scan (Table 2). The most common pathologies were traumatic subarachnoid hemorrhage (29%) followed by contusion (27%), epidural hematoma (25%), and acute subdural hematoma (19%). As expected, the prevalence of those pathologies was higher among ICU patients. A skull fracture was present in almost half of all patients in the admission and ICU strata. The median Marshall and Rotterdam scores for the entire cohort were 2 (IQR: 1–2) and 2 (IQR: 2–3), respectively.

**Table 2:** Details of initial brain CT imaging.

**Characteristic**	**Total**	**Admission stratum**	**ICU stratum**	***p*-value**
Contusion	58 (27%)	9 (10%)	49 (39%)	**<0.001**
Traumatic axonal injury	28 (13%)	6 (7%)	22 (18%)	**0.030**
Acute subdural hematoma	40 (19%)	8 (9 %)	32 (26%)	**0.003**
Subacute or chronic subdural hematoma	0 (0%)	0 (0%)	0 (0%)	-
Traumatic subarachnoid hemorrhage	62 (29%)	9 (10%)	53(42%)	**<0.001**
Epidural hematoma	32 (15%)	8 (9%)	24 (19%)	0.053
Intraventricular hemorrhage	21 (10%)	1 (1%)	20 (16%)	**<0.001**
Skull Fracture	103 (48%)	30 (33%)	73 (58%)	**<0.001**
Subdural collection density	0 (0%)	0 (0%)	0 (0%)	-
Mass lesion	9 (4%)	0 (0%)	9 (7%)	**0.023**
Cisternal compression	21 (10%)	1 (1%)	20 (16%)	**<0.001**
Midline shift	7 (3%)	0 (0%)	7 (6%)	0.057
Any intracranial abnormality	133 (62%)	33 (36%)	100 (80%)	**<0.001**
Marshall CT Score (IQR)	2 (1-2)	1 (1-2)	2 (2-2)	**<0.001**
Rotterdam CT Score (IQR)	2 (2-3)	2 (2-2)	2 (2-3)	**<0.001**

***CT**, Computed Tomography; **IQR**, Interquartile range. Significant differences (p-value of <0.05 in the Mann-Whitney U test for continuous variables and chi-squared test for categorical variables) between the admission and ICU stratum are printed bold*.

### Clinical Care and Outcome

The median length of stay for pediatric TBI patients was 2 (IQR 1-4) days in the admission stratum and 10 (IQR 5-24) days in the ICU stratum. An emergency intracranial surgery was performed in 15% of ICU patients while 18% underwent emergency extracranial surgery. One-third of all ICU patients required emergency or non-emergency intracranial surgery during their entire hospital stay (this included placement of intracranial pressure monitoring devices). This differed significantly from patients in the admission stratum where no emergency intracranial surgery (*p* < 0.001) and 2 (2%) emergency extracranial surgeries (*p* < 0.001) were performed. At six months follow-up, the mortality rate and rate of an unfavorable outcome of pediatric TBI patients in the ICU stratum were 5 and 16% respectively (Table 3). In the admission stratum, no patient died (mortality rate: 0%) and with 1% (1 patient), the rate of an unfavorable outcome was significantly lower compared to ICU patients (*p* = 0.001). Taken together, this yields a mortality rate of 3% and rate of an unfavorable outcome of 10% for the entire pediatric CENTER-TBI cohort in our study. Of the six patients with a fatal outcome, four died due to their initial head injury. In the remaining two patients, the exact cause of death was not documented. In multivariate analysis, 35 patients were excluded due to missing data. In the remaining pediatric TBI patients admitted to the regular ward or ICU, only total GCS as well as the occurrence of secondary insults were significant predictors for an unfavorable outcome six months after the injury (Table 4).

**Table 3:** Clinical status, hospital course, and outcome of pediatric TBI patients in the admission and ICU stratum of the CENTER-TBI core study.

**Characteristic**	**Total**	**Admission stratum**	**ICU stratum**	***p*-value**
AVPU				**<0.001**
- Alert	115 (51%)	80 (84%)	35 (27%)	
- Verbal	23 (10%)	12 (13%)	11 (8%)	
- Pain	20 (8%)	1 (1%)	19 (14%)	
- Unresponsive	60 (26%)	1 (1%)	59 (45%)	
- Unknown	9 (4%)	1 (1%)	8 (6%)	
GCS (IQR)	14 (10-15)	15 (15-15)	11 (6-14)	**<0.001**
GCS – motor (IQR)	6 (5-6)	6 (6-6)	5 (1-6)	**<0.001**
Pupillary response				**0.001**
- Both reactive	205 (92%)	92 (100%)	113 (86%)	
- One reactive	5 (2%)	0 (0%)	5 (4%)	
- Both unreactive	13 (6%)	0 (0%)	13 (10%)	
Vomiting	28 (14%)	17 (21%)	11 (10%)	**0.040**
Signs of facial fractures	16 (8%)	2 (3%)	14 (12%)	**0.030**
Facial contusion	19 (10%)	6 (8%)	13 (11%)	0.512
Signs of head/skull trauma	61 (31%)	14 (18%)	47 (41%)	**<0.001**
Signs of skull base trauma	21 (11%)	2 (3%)	19 (17%)	**0.004**
Alteration of Consciousness	35 (18%)	10 (13%)	25 (22%)	0.136
Loss of consciousness	76 (39%)	36 (45%)	40 (35%)	0.214
Seizures	6 (3%)	1 (1%)	5 (4%)	0.412
Post-traumatic amnesia <4 h	3 (2%)	3 (4%)	0 (0%)	0.136
Headaches	26(13%)	20 (25%)	6 (5%)	**<0.001**
Neurological deficit	16 (8%)	2 (3%)	14 (12%)	**0.030**
Length of stay	5 (2-13)	2 (1-4)	10 (5-24)	**<0.001**
Emergency intracranial surgery	20 (10%)	0 (0%)	20 (15%)	**<0.001**
Emergency extracranial surgery	25 (11%)	2 (2%)	23 (18%)	**<0.001**
Intracranial surgery	47 (21%)	3 (3%)	44 (33%)	**<0.001**
Extracranial surgery	45 (20%)	7 (7%)	38 (28%)	**<0.001**
GOSE at six months (IQR)	7 (6-8)	8 (7-8)	6 (5-8)	**<0.001**
Mortality	6 (3%)	0 (0%)	6 (5%)	0.099
Unfavorable outcome (GOSE 1-4) at 6 months	20 (10%)	1 (1%)	19 (16%)	**0.001**

***AVPU**, Awake, Verbal, Pain, Unresponsive; **GCS**, Glasgow Coma Scale; **GOSE**, Glasgow Outcome Scale Extended; **IQR**, Interquartile range. Significant differences (p-value of <0.05 in the Mann-Whitney U test for continuous variables and chi-squared test for categorical variables) between the admission and ICU stratum are printed bold*.

**Table 4:** Multivariate logistic regression analysis to unfavorable outcome (GOSE 1-4) in pediatric TBI patients in the admission and ICU stratum of the CENTER-TBI core study.

**Predictor**	**Beta**	***p*-value**	**OR (95% CI)**
Age	0.030	0.738	0.543 (0.020-11.529)
Gender	−0.884	0.248	1.030 (0.871-1.241)
RTI	0.472	0.599	0.413 (0.086-1.851)
GCS	−0.378	<0.001	0.686 (0.535-0.833)
Total ISS	0.014	0.625	1.014 (0.958-1.075)
Secondary insult	3.081	<0.001	21.782 (4.137-160.287)

***GCS**, Glasgow Coma Scale; **ISS**, Injury Severity Score; **RTI**, Road traffic incident; **OR**, Odds ratio; **CI**, Confidence interval. The p-value tests the hypothesis that the given variable has no association with unfavorable outcome at six months*.

### Mild TBI: Admission vs. ICU

Notably, 50 patients with mild TBI (GCS 13–15) were admitted to the ICU. Accidents in those patients were more commonly set in streets/highways when compared to patients with mild TBI in the admission stratum (58 vs. 39%; *p* = 0.034). While 81% of patients with mild TBI in the admission stratum had a GCS of 15 (median GCS: 15 (15–15), this was only the case in 52% of patients with mild TBI that were admitted to the ICU (median GCS: 15 (14–15; *p* < 0.001). Prevalence of concurrent injuries (e.g., abdominal injuries: 11% (ICU) vs. 2% (admission); *p* = 0.109) was higher in the mild TBI patients in the ICU compared to the regular ward and the ISS was significantly higher in those patients as well (21 (16–57) vs. 16 (9–22); *p* < 0.001). At six months follow-up, the median GOSE was 8 (7–8) in the admission stratum vs. 7 (6–8) in the ICU stratum (*p* = 0.001).

### Severe TBI: Favorable vs. Unfavorable Outcome

**Figure 4:**
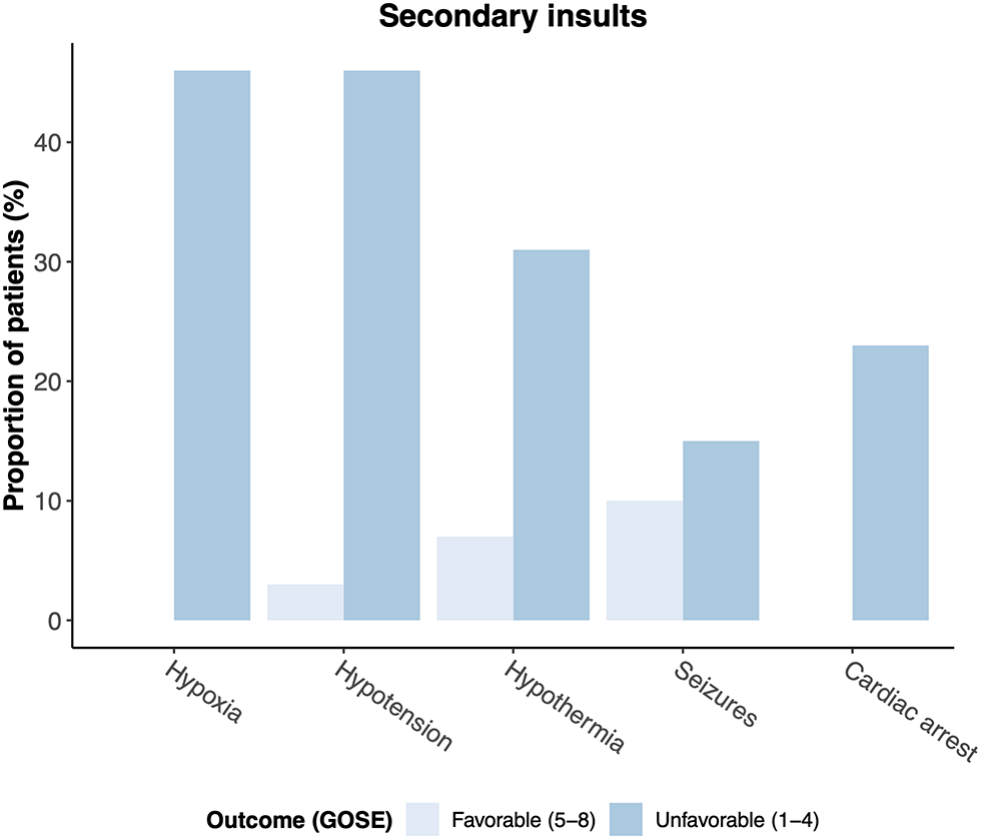
Secondary insults at admission in patients with favorable (GOSE 5–8) and unfavorable (GOSE 1–4) outcome at six months.

Severe TBI (GCS 3–8) occurred in 46 patients all of whom were subsequently admitted to the ICU. An unfavorable outcome was reported in 13 of 43 patients (30%) with severe TBI and available GOSE at 6 months (Table 5). Patients with severe TBI and an unfavorable outcome were considerably more often involved in road traffic incidents compared to patients with favorable outcome (69 vs. 53%; *p* = 0.332). Median GCS at presentation was lower in the unfavorable outcome group (4 (3–6) vs. 6 (3–7); *p* = 0.157) and bilateral unreactive pupillary response was documented in almost one-third of patients. Secondary insults at presentation, while rather rare in the group with favorable outcomes, were significantly more common in the group with unfavorable outcomes (Figure 4, Table 5).

**Table 5:** Comparison of pediatric severe TBI patients with favorable or unfavorable outcome in the CENTER-TBI core study.

**Characteristic**	**Total**	**Favorable outcome (*n* = 185)**	**Unfavorable outcome (*n* = 20)**	***p*-value**
Age (IQR)	15 (9-16)	15 (8-16)	15 (10-17)	0.717
Sex				0.108
- Female	17 (37%)	7 (23%)	7 (54%)	
- Male	29 (63%)	23 (77%)	6 (46%)	
Injury cause				0.056
- Road traffic incident	28 (61%)	16 (53%)	9 (69%)	
- Incidental fall	10 (22%)	10 (33%)	0 (0%)	
- Other non-intentional injury	7 (15%)	4 (13%)	3 (23%)	
- Violence	0 (0%)	0 (0%)	0 (0%)	
- Other/Unknown	1 (2%)	0 (0%)	1 (8%)	
Injury road incident				0.930
- Motor vehicle occupant	7 (25%)	4 (25%)	2 (22%)	
- Pedestrian	7 (25%)	3 (19%)	3 (33%)	
- Cyclist	6 (21%)	3 (19%)	2 (22%)	
- Scooter	3 (11%)	2 (13%)	1 (11%)	
- Motor Bike	4 (14%)	3 (19%)	1 (11%)	
- Other	1 (4%)	1 (6%)	0 (0%)	
Safety helmet (cyclist, scooter, motor bikers)				0.301
- Yes	6 (46%)	4 (50%)	2 (50%)	
- No	6 (46%)	4 (50%)	1 (50%)	
- Unknown	1 (8%)	9 (0%)	1 (25%)	
Total ISS (IQR)	34 (25-48)	29 (21-49)	41 (34-57)	**0.008**
GCS (IQR)	5 (3-7)	6 (3-7)	4 (3-6)	0.157
GCS motor (IQR)	2 (1-4)	3 (1-5)	2 (1-4)	0.694
Pupillary response				0.342
- Both reactive	36 (78%)	25 (83%)	9 (69%)	
- One reactive	2 (4%)	1 (3%)		
- Both unreactive	8 (17%)	4 (13%)	4 (31%)	
Secondary Insult: Hypoxia	7 (15%)	0 (0%)	6 (46%)	**<0.001**
Secondary Insult: Hypotension	7 (15%)	1 (3%)	6 (46%)	**0.001**
Secondary Insult: Cardiac arrest	3 (7%)	0 (0%)	3 (23%)	**0.038**
Secondary Insult: Hypothermia	6 (13%)	2 (7%)	4 (31%)	**0.008**
Secondary Insult: Seizures	5 (11%)	3 (10%)	2 (15%)	0.836
Length-of-stay (IQR)	28 (10-43)	28 (10-42)	29 (6-59)	0.814
GOSE at 6 months (IQR)	6 (4-7)	7 (6-8)	3 (1-3)	**<0.001**

***GCS**, Glasgow Coma Scale; **GOSE**, Glasgow Outcome Scale Extended; **ISS**, Injury Severity Score; **IQR**, Interquartile range. Significant differences (p-value of < 0.05 in the Mann-Whitney U test for continuous variables and chi-squared test for categorical variables) between the favorable and unfavorable outcome group are printed bold*.

### Comparison With the CENTER-TBI Registry Dataset

The CENTER-TBI registry was used to compare the 227 pediatric TBI patients in the core dataset with a substantially larger cohort of 687 pediatric TBI patients in the registry dataset and confirm key findings regarding injury causes and severity (Table 6). Pediatric patients had been registered in 46 of the 59 CENTER-TBI registry participants. The median age in pediatric TBI patients within the registry dataset was 12 (IQR 4-16, range 0–17) years and 64% of patients were males. The fraction of patients in the admission stratum was considerably higher (62%) compared to the core dataset (42%). Corresponding to the results from the core dataset, streets were the most common injury place (42%), followed by injuries at home (28%). Likewise, road-traffic incidents (41%) and falls (40%) were the most common causes of pediatric TBI in the CENTER-TBI registry. In road-traffic incidents, pediatric patients were most commonly involved as cyclists or pedestrians (together 61%). Total ISS scores were comparable to those obtained in the core dataset and amounted to 10 (5–13) in the admission stratum and 25 (16–38) in the ICU stratum, underlining the close association of pediatric TBI to concurrent injuries especially in the ICU setting. Similar to the results from the core dataset, brain injury was classified as serious to severe in most cases (AIS 3 (2–4) ). GCS ratings were also similar to the CENTER-TBI core study with a GCS of 15 (14–15) in the admission stratum and 12 (7–15) in the ICU stratum. On brain CT, an abnormality was detected in 49% of pediatric TBI patients in the registry dataset.

**Table 6:** Comparison of patient characteristics between the CENTER-TBI core vs. registry datasets.

**Characteristic**	**Total**		**Admission stratum**		**ICU stratum**	
	**Core**	**Registry**	**Core**	**Registry**	**Core**	**Registry**
Number of patients	227	687	95	423	132	264
Age (IQR)	14 (8-16)	12 (4-16)	13 (9-16)	12 (5-16)	14 (8-16)	12 (4-16)
Sex						
- Female	81 (36%)	246 (36%)	32 (34%)	147 (35%)	49 (37%)	99 (38%)
- Male	146 (64%)	441 (64%)	63 (66%)	276 (65%)	83 (63%)	165 (62%)
Injury Place						
- Street	118 (52%)	298 (42%)	38 (40%)	169 (38%)	80 (61%)	129 (49%)
- Home	40 (18%)	192 (28%)	19 (20%)	126 (30%)	21 (16%)	66 (25%)
- Work/School	14 (6%)	4 (1%)	7 (7%)	3 (1%)	7 (5%)	1 (0%)
- Sport	40 (18%)	72 (10%)	21 (22%)	56 (13%)	19 (14%)	16 (6%)
- Public	11 (5%)	96 (14%)	8 (8%)	69 (4%)	3 (2%)	36 (14%)
- Other	2 (1%)	31 (5%)	0 (0%)	16 (4%)	2 (2%)	15 (6%)
- Unknown	2 (1%)	3 (0%)	2 (2%)	2 (0%)	0 (0%)	1 (0%)
Injury Cause						
- RTI	110 (48%)	282 (41%)	33 (35%)	152 (36%)	77 (58%)	130 (49%)
- Fall	78 (34%)	276 (40%)	41 (43%)	184 (43%)	37 (28%)	92 (35%)
- Other	39 (17%)	129 (19%)	21 (22%)	87 (21%)	18 (14%)	42 (16%)
GCS (IQR)	14 (10-15)	15 (13-15)	15 (15-15)	15 (14-15)	11 (6-14)	12 (7-15)
GCS – motor (IQR)	6 (5-6)	6 (6-6)	6 (6-6)	6 (6-6)	5 (1-6)	6 (4-6)
AIS Brain Injury (IQR)	4 (3-4)	3 (2-4)	3 (3-3)	2 (1-3)	4 (4-5)	4 (3-5)
ISS (IQR)	18 (10-32)	13 (9-22)	10 (9-17)	10 (5-13)	26 (17-41)	25 (16-38)
Pupillary response						
- Both reactive	205 (92%)	619 (94%)	92 (100%)	403 (99%)	113 (86%)	216 (85%)
- One reactive	5 (2%)	20 (3%)	0 (0%)	5 (1%)	5 (4%)	15 (6%)
- Both unreactive	13 (6%)	23 (3%)	0 (0%)	0 (0%)	13 (10%)	23 (9%)
CT Brain: Any intracranial abnormality	133 (59%)	318 (49%)	33 (35%)	115 (30%)	100 (76%)	203 (77%)

***AIS**, Abbreviated Injury Scale; **CT**, Computed Tomography; **GCS**, Glasgow Coma Scale; **ISS**, Injury Severity Scale; **IQR**, Interquartile range; **RTI**, Road-traffic incident*.

## Discussion

Traumatic brain injury in children is a very serious condition that can lead to lifelong disability but still presents as a scientific field with a general lack of research and evidence (6). Furthermore, due to particularities in the pathophysiology of pediatric TBI with e.g., different absorption of traumatic forces and different dynamics of head acceleration, it is precarious to transfer findings from adult TBI patients to the pediatric population (2). In an effort to contribute to a better understanding of injury characteristics and clinical care of pediatric TBI, we, therefore, analyzed the multi-center, prospectively collected CENTER-TBI core and registry datasets for patients younger than 18 years with TBI who were admitted to the hospital ward or ICU. Our key findings in the core study could be confirmed in the larger registry dataset, supporting the external validity of our results.

Road traffic incidents were the most common injury causes overall (admission and ICU stratum) ahead of incidental falls, which is in line with previous reports (7, 8). While road traffic incidents were especially prominent in the ICU stratum and associated with severe injuries, an association that can also be seen in previous studies (9, 10), incidental falls were the most common injury cause in the admission stratum. This differs from the overall, predominately adult CENTER-TBI patient population where incidental falls were the most common cause of TBI. However, similarly to our findings in the pediatric cohort, road-traffic incidents also were the most common cause of TBI in ICU patients (11). Pediatric patients in road traffic incidents were most commonly involved as pedestrians. When children were involved as cyclists, scooter drivers or motor bikers, more than half of them did not wear a safety helmet which is an important and noteworthy finding in regard to possible preventive targets and efforts.

Brain CT imaging in the young patients in our analysis allowed insights into common pathologies behind pediatric TBI. The most common finding hereby was traumatic subarachnoid hemorrhage which is a relevant diagnosis in children as its complications include hydrocephalus and cerebral vasospasms (12). Other common findings were epidural and acute subdural hematomas. Skull fractures were remarkably present in nearly half of the pediatric patient cohort. Those findings correspond well to the numbers from previous studies that in summary reported skull fractures and cerebral contusions as the most common CT abnormalities (3). In 40% of patients in our study, no intracranial abnormality could be detected on the initial brain CT.

Because TBI patients within the CENTER-TBI study were stratified into different strata upon enrollment, analysis of the data offers the opportunity to directly compare pediatric TBI patients treated in different hospital settings, in particular patients treated on the regular ward vs. the ICU. We found, as expected, considerable differences in injury cause and severity between the admission and ICU stratum. While patients requiring intensive care were more often involved in road-traffic incidents, incidental falls were the most common cause in patients in the admission stratum. Injury severity was in general significantly higher in ICU patients, as indicated by higher ISS and GCS scores, and prevalence of concurrent injuries in other body regions, especially thorax and abdomen, was greater. Notably, more than three quarters of patients also suffered from other body injuries, emphasizing the importance of a general and extensive clinical examination in pediatric TBI patients. A total of 61% of pediatric patients in our cohort were classified as mild TBI. Interestingly, 36% of them were admitted to the ICU. While this finding corresponds to the number of mild TBI patients admitted to the ICU in the adult CENTER-TBI patient population (36%), discussed reasons such as advanced age, comorbidities or antithrombotic drugs which might increase the risk for lesion progression are not applicable for the pediatric patients assessed in our study (11). Rather, we found a higher prevalence of road-traffic incidents with more concurrent injuries in other body parts in affected mild TBI patients as well as an increased presence of neurological deficits which could be possible explanations for the physician's decision in those cases. Moreover, the high rate of extra- and intracranial surgeries in general might have warranted observation of pediatric mild TBI patients on the ICU in some cases. Because participating centers within the CENTER-TBI study were not generally specialized in treating pediatric TBI, overtreatment cannot be ruled out and might have additionally affected the high ICU admission rate of pediatric mild TBI patients.

Severe TBI was present in 22% of all patients in our cohort of pediatric TBI. As expected, all of those patients were treated in the ICU with an injury so grave that emergency intracranial surgery had to be performed in 4 of 46 patients (9%). An unfavorable outcome was reported in 30% of pediatric severe TBI patients in our cohort, showing associations with road traffic incidents, lower GCS at presentation as well as the occurrence of secondary injury insults such as hypotension at admission. The high prevalence of road-traffic incidents in children with severe TBI and an unfavorable outcome emphasizes the need and potential for more preventive efforts.

Considering the overall outcome in our cohort of pediatric TBI patients, a GOSE score of 7 or 8 could be observed in 64% of cases. However, an unfavorable outcome (GOSE 1-4) was still present in 10% of pediatric TBI patients which is comparable to numbers from single-center studies reported from India (10%) and the United States (16%) (13, 14). Independent predictors for an unfavorable outcome six months after TBI in pediatric patients who were admitted to the regular ward or ICU were GCS and the occurrence of secondary insults in multivariate analysis. Secondary systemic insults such as hypoxia and hypotension were significantly more common in patients with unfavorable outcome which seems to confirm previous studies from both pediatric and adult patient cohorts (15–22). Importantly, these physiological parameters impose potential treatment targets: Active airway management such as early intubation and early tracheostomy have been shown to be associated with better outcomes in adults (23–26). Similarly, the relationship between low preadmission blood pressures and mortality is well established and recent results from large multi-center trials suggest to consider notably higher blood pressure targets than the >90 mmHg systolic blood pressure threshold stated in current guidelines (27, 28). Future studies examining blood pressure treatment levels in both adult and pediatric patients are needed to find optimal treatment thresholds.

Despite a relatively high injury severity, mortality was still rather low in the overall pediatric CENTER-TBI cohort assessed in our study (3%) as well as in the group of pediatric severe TBI patients (9%) and thus lower than in the overall CENTER-TBI cohort that includes predominately adult patients (14.9% for patients admitted to the regular ward or ICU) (11). Similar results for pediatric TBI have been reported in several comparable studies, indicating the great potential for recovery in children, even when presenting with severe TBI (1, 7, 29, 30). In the literature, differences in outcome between pediatric and adult TBI patients have been explained by reasons such as a greater flexibility of cranial bones in young children, providing a higher capacity of traumatic force absorption (31). Although research on this specific field is still very limited, lower mortality in the pediatric population of the CENTER-TBI study compared to the adult population might be generally related to the immense disparity in the presence of comorbidities, frailty or antithrombotic medication.

Nevertheless, alarming numbers have very recently been published concerning age-adjusted TBI mortality for patients aged 0–19 years in the US: Despite an initial decline from 1999 to 2012, pediatric TBI mortality has been raising again since 2013 (32). And although functional outcomes early after TBI might be better in children, there is growing evidence that they are more vulnerable to long-term cognitive deficits compared to adults (33). Therefore, and as disabilities and deficits are a huge burden in surviving patients with pediatric TBI, every effort should be made to prevent the occurrence of this condition.

With the findings in the CENTER-TBI core study and their validation in the CENTER-TBI registry, our analysis displays the most common injury causes of pediatric TBI in Europe at present. Also, it confirms known predictors for an unfavorable outcome after TBI in children. While neurosurgeons, pediatricians, and other health practitioners should be especially aware of the risks associated with secondary insults, legislators are reminded that further preventive efforts such as advertising the use of safety helmets or building safer infrastructure might still be needed to reduce the incidence of severe TBI.

## Conclusion

TBI in pediatric patients within the CENTER-TBI study that were admitted to the regular ward or ICU were most commonly caused by road-traffic incidents and incidental falls. Injury severity was serious especially in ICU patients and concurrent injuries in other body parts were common. GCS and the occurrence of secondary insults were identified as predictors for an unfavorable outcome (GOSE 1–4) at six months follow-up. The current analysis suggests, that preventive efforts could still be very effective in decreasing the incidence of TBI.

## Limitations

There are several limitations to this analysis. Recruitment to the CENTER-TBI core study was conducted at the discretion of the participating centers and thus influenced by local logistics and academic interests which might be a potential source of selection bias. Because of data anonymization reasons, no information on the specific countries where the TBI patients were recruited can be obtained from the CENTER-TBI database. The data used for our analyses might, therefore, be derived from only a subset of countries and the results should be generalized with caution. Moreover, although the CENTER-TBI study included pediatric as well as adult patients, the participating centers were mainly general hospitals and not specialized pediatric centers and might thus not have primarily managed pediatric TBI in some regions or countries. Because data on the type of ICU is not available within the CENTER-TBI database, a possible bias from the treatment of pediatric patients in specialized ICUs cannot be ruled out. In addition, not all participating centers enrolled pediatric patients. This might be the reason why pediatric TBI was underrepresented in comparison to adult TBI in the CENTER-TBI study. While we can therefore not comment on the absolute incidence of pediatric TBI in Europe, we still provide a large multi-center pediatric patient cohort that can give important insights into injury causes and patterns. Furthermore, although the data were prospectively collected, missing data was still present especially regarding to long-term outcomes with GOSE ratings being only available for 90% of patients.

## Data Availability Statement

The datasets generated for this study are available on request to the corresponding author and with permission of the CENTER-TBI management committee.

## Ethics Statement

The CENTER-TBI study is compliant with all relevant EU- and national laws of recruiting centers in regard to privacy, data protection and ethical standards and in accordance with the Declaration of Helsinki (“Ethical Principles for Medical Research Involving Human Subjects”). Informed consent was obtained from all patients and/or their legal representatives, according to the local legislations, included in this study. Ethical approval was obtained for each recruiting site (see https://www.center-tbi.eu/project/ethical-approval for the list of sites, ethical committees, approval numbers and approval dates).

## Author Contributions

LR and AY designed the study, conducted the data analysis, interpreted the data, and co-wrote the manuscript. KZ, AU, and AE helped with data interpretation and critically revised the manuscript.

## Conflict of Interest

The authors declare that the research was conducted in the absence of any other commercial or financial relationships that could be construed as a potential conflict of interest.
